# First cases of *Angiostrongylus cantonensis* infection reported in Martinique, 2002–2017

**DOI:** 10.1051/parasite/2020032

**Published:** 2020-05-12

**Authors:** Céline Dard, Eve Tessier, Duc Nguyen, Loïc Epelboin, Dorothée Harrois, Christopher Swale, André Cabié, Katia de Meuron, Charline Miossec, Nicole Desbois-Nogard

**Affiliations:** 1 Laboratoire de Parasitologie-Mycologie, Centre Hospitalier Universitaire Grenoble Alpes, CS 10217 38043 Grenoble Cedex 9 France; 2 Institute for Advanced Biosciences (IAB), INSERM U1209 – CNRS UMR5309, Université Grenoble Alpes 38700 La Tronche France; 3 Laboratoire de Parasitologie-Mycologie, Centre Hospitalier Universitaire de Martinique Fort-de-France 97200 Martinique France; 4 Service des Maladies Infectieuses et Tropicales, Centre Hospitalier Universitaire de Martinique Fort-de-France 97200 Martinique France; 5 Service des Maladies Infectieuses et Tropicales, Centre Hospitalier Andrée Rosemon Cayenne 97300 Guyane France; 6 Laboratoire de Biologie Médicale, Centre Hospitalier de Basse-Terre Basse-Terre 97100 Guadeloupe France; 7 INSERM CIC 1424, Centre Hospitalier Universitaire de Martinique Fort-de-France 97200 Martinique France; 8 Université des Antilles, EA7524 97200 Martinique France; 9 Maison de la Femme, de la Mère et de l’Enfant, Centre Hospitalier Universitaire de Martinique Fort-de-France 97200 Martinique France

**Keywords:** *Angiostrongylus cantonensis*, Angiostrongyliasis, Eosinophilia, Helminth, Meningitis, Encephalitis, Caribbean, Martinique

## Abstract

Neuroangiostrongyliasis is a parasitic disease caused by the accidental ingestion of the nematode *Angiostrongylus cantonensis* in its larval form. Human infection can lead to eosinophilic meningitis, sometimes complicated by life-threatening radiculomyelitis or encephalitis. Although some cases have been reported from other Caribbean Islands, no cases have been diagnosed in Martinique so far. Here, we report the first eight laboratory-confirmed cases of neuroangiostrongyliasis on the island of Martinique, French West Indies, between 1 January 2002 and 31 December 2017. One case was fatal and five resulted in neurological sequelae. The medical community should consider the risk of *A. cantonensis* infection in patients living in or returning from Martinique.

## Abstract (fr)

L’angiostrongylose neuroméningée est une maladie parasitaire causée par l’ingestion accidentelle du nématode *Angiostrongylus cantonensis* sous sa forme larvaire. L’infection humaine peut conduire à une méningite à éosinophiles, pouvant évoluer en radiculomyélite ou encéphalite menaçant le pronostic vital. Bien que des cas aient été rapportés dans d’autres îles des Caraïbes, aucun cas n’avait été diagnostiqué à la Martinique jusqu’à présent. Dans cet article, nous caractérisons les huit premiers cas d’angiostrongylose neuroméningée biologiquement diagnostiqués à la Martinique, Antilles françaises, entre le 1^er^ janvier 2002 et le 31 décembre 2017. Un cas s’est révélé mortel et cinq ont engendré des séquelles neurologiques. La communauté médicale doit désormais considérer le risque d’infection à *A. cantonensis* chez les patients vivant ou revenant d’un voyage à la Martinique.

## Introduction

*Angiostrongylus cantonensis* is a nematode parasite that is the leading cause of infectious eosinophilic meningitis in humans in tropical and sub-tropical regions [6]. The life cycle involves rats as definitive hosts (mainly *Rattus* spp.) [49], various gastropods as intermediate hosts, and crustaceans [23], fishes and various other species as paratenic hosts [6]. Human infection is accidental, by ingestion of stage 3 larvae (L3) in gastropods or in paratenic hosts [11]. Neuroangiostrongyliasis is commonly a self-limited meningitis syndrome, but a large spectrum of clinical manifestations is possible [5]. Clinical manifestations range from asymptomatic disease and mild headaches to radiculomyelitis and encephalitis that can lead to permanent neurological injury or even death [29]. Most human cases of neuroangiostrongyliasis have been recorded in East and Southeast Asia, and the Pacific Basin, but the disease appears to be emerging in Australia [1], South America [13, 32, 44], the United States [3, 21], and some islands of the Caribbean (Cuba, Haiti, Dominican Republic, Jamaica, and Guadeloupe) [12, 19, 42]. Several patients infected with *A. cantonensis* have been diagnosed with neuroangiostrongyliasis – but not reported in the scientific literature – during the last few years on the island of Martinique, a French overseas department in the Lesser Antilles with a population of 371,200 inhabitants as of 1 January 2018 (INSEE census, French National Institute of Statistics and Economic Studies, https://www.insee.fr). The objectives of this study were to estimate the incidence and assess the clinical and biological features of neuroangiostrongyliasis in Martinique.

## Patients and methods

### Study design

A retrospective single-centre observational study was undertaken at the University Hospital of Martinique – the main hospital on the island – spanning the period 1 January 2002 – 31 December 2017.

### Inclusion and exclusion criteria

The following clinical and biological inclusion criteria were used: (i) neurological symptoms requiring lumbar puncture; (ii) eosinophilic meningitis defined as the presence of more than 10 eosinophils/mm^3^ in the cerebrospinal fluid (CSF) or ≥10% of the total CSF leukocyte count [5]; and (iii) detection of anti-*A. cantonensis* immunoglobulins in serum and/or CSF by indirect immunofluorescence assay (IIF) [22] or by western-blot (31-kDa antigen [34]). Patients with eosinophilia in the CSF due to a traumatic lumbar puncture or blood eosinophilia of another aetiology were excluded. Neuroangiostrongyliasis cases were defined as “confirmed” for patients who met the first two inclusion criteria, and with the detection of specific anti-*A. cantonensis* immunoglobulin*s* in serum and/or CSF by western-blot. When *A. cantonensis* serology was found to be positive by IIF only, cases were defined as “probable” because of the low/lack of specificity of this method for nematode infections, in which some cross-reactions can be observed [47,48].

### Data collection

Demographic data including exposure history, laboratory results (from blood and CSF), clinical presentation, imaging features (brain scan or MRI), and outcomes (recovery, sequelae, and death) were retrospectively collected from the medical charts, according to the legal and ethical guidelines of the French National Committee on Data Protection (CNIL).

### *Angiostrongylus cantonensis* serology by indirect immunofluorescence assays

From 2002 to 2010, samples provided for *A. cantonensis* serological testing were sent to the only laboratory performing neuroangiostrongyliasis infection diagnosis in France, including its overseas regions (Laboratory of the Centre Hospitalier de Gonesse, Gonesse, France). An indirect immunofluorescence (IIF) assay was used for the detection of antibodies against *A. cantonensis* antigens, as described in [18]. Due to logistical constraints and availability of certain reagents, patient serodiagnosis in this laboratory ceased in 2010 and no other laboratory then performed the test in France. Subsequent serological analyses were therefore performed in Thailand or Switzerland by western-blot.

### *Angiostrongylus cantonensis* serology by western-blot

Western blot assays using antigens derived from *A. cantonensis* adult worms were performed to detect IgG against *A. cantonensis* in either the Department of Parasitology, Faculty of Medicine Siriraj Hospital, Mahidol University, Bangkok, Thailand, or the Swiss Tropical and Public Health Institute, Basel, Switzerland. The detection of the 31-kDa band confirmed serum positivity as it shows high sensitivity and specificity (>99% for both) for the diagnosis of *A. cantonensis* infections [17].

## Results

Descriptive results of the clinical presentations, including biological, imaging, and epidemiological features, are shown in Table 1 and statistically analyzed in Table 2. During the 16-year period of the study, four confirmed and four probable cases of neuroangiostrongyliasis were diagnosed in Martinique, among which three were children below 2 years of age, one was an 11-year-old boy, and four were adults aged from 37 to 64 years. The annual incidence rate was 0.14 cases/100,000 inhabitants/year (95% CI [0.04–0.23]) with six of the cases occurring during the rainy season from June to November. All patients were born and lived in Martinique and none reported recent travel. Contact with molluscs was reported in two cases. All five patients older than 2 years of age (Table 1) presented with pre-existing mental disorders (pica syndrome, bipolar disorder, intellectual disability, or autism), which may have promoted the accidental or even deliberate consumption of snails. All cases presented with acute neurological signs and/or symptoms requiring a lumbar puncture: dysfunction of the cranial nerves (highlighted by clinical neurological examination of the 12 pairs of cranial nerves), headaches, axial hypotonia, seizures, radiculalgia, and neck stiffness. Clinical examination was difficult for one patient because of autism. During hospitalisation, five patients had fever and three had digestive symptoms (vomiting, abdominal pain, loss of appetite, and/or diarrhoea). Brain imaging was performed for all patients (CT scan or MRI): five presented abnormalities, with abnormal enlargement of the cerebral ventricles or cortical atrophy, and three were normal. Blood eosinophilia at admission was inconsistent, with a median of 1.72 G/L (13% of the WBC count in blood) and a range of 0.49–6.43 G/L (5–31%). Median eosinophilia in the CSF at first lumbar puncture was 74.5 (25% of the WBC count in CSF), with a range of 0–1550/mm^3^ (4–68%) and the maximum values during hospitalisation were 373.5 (54.5%), with a range of 45–1550/mm^3^ (18–68%). Most patients were treated with albendazole [35] and/or corticosteroids [18]. Clinical outcomes ranged from rapid recovery without sequelae for two patients to neurological sequelae manifested as strabismus and intellectual disability for five patients. One case was fatal for a 58-year-old man. Diagnosis was made by anti-*A. cantonensis* antibody detection in sera for seven patients and confirmed positive in CSF for five of them. One patient only showed detectable anti-*A. cantonensis* antibodies in the CSF. Serological analysis was negative for two patients nine days after admission, but positive 19–38 days after admission.

**Table 1:** Description of the probable cases (numbers 1–4) and confirmed cases (numbers 5–8) of *Angiostrongylus cantonensis* infection in Martinique, including clinical, biological, imaging, and epidemiological features.

Case	1	2	3	4	5	6	7	8
Demographic characteristics								
Year	2002	2002	2006	2008	2013	2015	2015	2015
Season	Dry	Rainy	Rainy	Rainy	Rainy	Dry	Rainy	Rainy
Sex	F	M	F	M	M	M	M	M
Age	10.5 months	11 months	13 months	37 years	64 years	61 years	58 years	11.5 years
Residential locality	Fort-de-France	Le Lamentin	Cap Ferré, Saint-Anne	Le Diamant	ND	Fort-de-France	Quartier Morne Etoile, le Lorrain	Quartier Petit Versailles, Saint-Anne
Medical history	None	None	None	Substance addiction, bipolar	Intellectual disability	Diogenes syndrome	Schizophrenia	Autism, stunted growth
Risky behaviour	Playing on soil	Accidental slug ingestion	Unknown	Undercooked snail consumption	Stays in forest several days	Unknown	Pica disorder	Pica disorder
Clinical presentation								
Fever	Yes	Yes	Yes	No	Yes	No	No	Yes
Neurological signs and symptoms	Strabismus, cranial nerves VI palsy	Seizures	Hypotonia, tetraplegia	Radiculalgia, cranial nerves VI palsy	Headaches, coma	Headaches, Neck stiffness, cranial nerves VI palsy	Extrapyramidal syndrome, seizures, left hemiparesis, coma	Difficult examination because of autism
Digestive signs and symptoms	Anorexia	None	None	Nausea, vomiting	None	Loss of appetite	None	Loss of appetite, constipation
Others	ND	ND	ND	ND	ND	ND	ND	Itching
Laboratory tests								
CRP (mg/L)	<5	<5	<5	11.6	74	<5	<5	<5
Total WBC count (G/L) D_0_	8.24	15.09	10.03	10.00	12.20	16.35	6.61	20.75
Eosinophils in blood G/L (%) D_0_/max value	0.49 (5)/5.46 (32)	2.23 (14)/3.23 (26)	2.11 (21)/3.66 (17)	0.62 (8)/0.89 (10)	1.40 (11)/2.49 (21)	1.77 (11)/2.76 (13)	1.67 (25)/1.98 (36)	6.43 (31)/6.43 (31)
WBC count in CSF 1st LP	170	600	0	389	1040	410	2280	1080
Eosinophils in CSF/mm^3^(%) 1st LP/max value	59 (35)/355 (48)	90 (15)/392 (49)	0 (0)/220 (38)	0 (0)/59 (62)	600 (60)/600 (60)	16 (4)/45 (18)	1550 (68)/1550 (68)	691 (64)
Proteinorachia g/L D_0_/max value	1.25/1.25	0.52/0.85	0.33/0.83	1.17/1.17	1.10/1.10	1.71/1.71	1.50/3.70	0.63
Glycorachia mmol/L D_0_/min value	0.1/0.1	1.7/1.0	3.5/0.2	2.3/1.8	4.3/4.3	1.0/1.0	3.3/0.9	3.4
Intracranial Hypertension	ND	ND	No	Yes, 37	No	Yes	ND	ND
Angiostrongylus serodiagnosis	Positive^a^ (D_13,_ D_28_, D_49_)	Negative^a^ (D_9_)	Negative^a^ (D_9_)	Negative^a^ D_24_	Positive D_3_, D_19_^b^	Positive^c^	Positive^c^	Positive^c^
		Positive^a^ (D_38_)	Positive^a^ (D_19_)					
Cross-reactions (sera)	Cysticercosis (1/2048e)	None	None	*Echinococcus*	*Toxocara*	*Toxocara, Filaria, Strongyloides*	*Toxocara, Strongyloides*	*Toxocara, Echinococcus, Strongyloides*
Specific antibodies in CSF	Positive^a^ (D_49_)	Positive^a^ (D_38_)	Positive^a^	Positive^a^ (D_24_)	ND	ND	Positive^b^ (D_8_)	Positive^b^
Parasitological examination of faeces	Negative	Negative	Negative	Negative	Negative	Negative	Negative	Negative
Brain CT-scan	Subnormal	None	None	ND	Normal	Normal	ND	Normal
Brain MRI	Ventricular dilatation	Normal	Hydrocephalus, ventricular dilatation, myelitis	Normal	Leukoaraiosis, cortico – subcortical atrophy	Normal	Ventricular dilatation	Abnormal(no precisions)
Management & outcome								
Treatment	Thiabendazole, albendazole	Albendazole	Albendazole, prednisone	Subtractive LP	Albendazole, prednisone	Albendazole + MPS	Albendazole + MPS	Albendazole + MPS
	Subtractive LP	Subtractive LP	Subtractive LP		Subtractive LP			
Hospitalization time (days)	55	15	34	19	49	66 + 90 days of physiotherapy	20	16
Clinical outcome, sequelae	Strabismus	Recovery	Psychomotor retardation	Strabismus	Recovery	Memory disorders, cranial nerves VI palsy	D_20_: death	Vision disorders

*Abbreviations*: CSF, cerebrospinal fluid; CT-scan, computerised tomography scan; CRP, C-reactive protein; Dx, day x after admission to hospital; LP, lumbar puncture; MPS, methylprednisolone; ND, not determined.

Normal values: CRP: <5 mg/L; protein level in CSF: 0.15–0.40 g/L; glucose level in CSF: 2.8–4.5 mmol/L; intracranial tension: <20.

a Serology performed by indirect immunofluorescence in the Medical Center of Gonesse, France.

b Serology performed by western-blot analysis in the Department of Parasitology, Faculty of Medicine Siriraj Hospital, Mahidol University Bangkok, Thailand.

c Serology performed by western-blot analysis in the Swiss Tropical and Public Health Institute, Basel, Switzerland.

**Table 2:** Characteristics of the eight patients with eosinophilic meningitis caused by *Angiostrongylus cantonensis*.

Characteristic	Result
Demographic characteristics	
Age (years)	24.3 [0.87–63.6]
Sex (male)	6 (75%)
Rainy season	6 (75%)
Exposure risk	
Reported contact with snails	2 (25%)
Previous mental disorders	5 (63%)
Clinical picture	
Fever (>38 °C)	5 (63%)
Digestive signs and symptoms	3 (38%)
Neurological signs and symptoms	8 (100%)
Headaches	2 (25%)
Neck stiffness	1 (13%)
Dysfunction of cranial nerves	3 (38%)
Seizure	2 (25%)
Axial hypotonia, hemiparesia	2 (25%)
Radiculalgia	1 (13%)
Coma	2 (25%)
Brain imaging	
Normal	3 (38%)
Enlargement of cerebral ventricles	3 (38%)
Cortical atrophy	1 (13%)
Laboratory results	
In blood	
C-reactive protein > 5 mg/L	2 (25%)
Total WBC count (G/L)	10.0 [6.61–20.75]
Blood eosinophilia at admission (G/L)	1.72 [0.49–6.43]
Blood eosinophilia at admission (% of WBC)	12.5 [5–31]
Max blood eosinophilia during hospitalisation (G/L)	2.99 [0.89–6.43]
Max blood eosinophilia during hospitalisation (% of WBC)	23.5 [10–36]
*A. cantonensis* positive serodiagnosis	7 (88%)
In CSF	
Eosinophilia in CSF at first LP (/mm^3^)	74.5 [0–1550]
Eosinophilia in CSF at first LP (% of WBC)	25.0 [0–68]
CSF glucose at first LP (mmol/L)	2.81 [0.1–4.3]
Protein level in CSF at first LP (g/L)	1.14 [0.33–1.71]
Protein level > 0.45 g/L at first LP	7 (88%)
Presence of antibodies in CSF (among those tested)	6 (100%)
Management	
Length of hospital stay (days)	27 [15–66]
Subtractive LP	5 (63%)
Corticosteroids	5 (63%)
Anthelmintic therapy	7 (88%)
Outcome	
One year recovery	2 (25%)
One year neurological sequelae	5 (63%)
One year mortality	1 (13%)

*Abbreviations*: CSF, cerebrospinal fluid; LP, lumbar puncture; WBC, white blood cell.

Descriptive results are presented as *n* (%) and as median (min–max).

## Discussion

This study reports the first eight laboratory-confirmed cases of neuroangiostrongyliasis in Martinique, which occurred between 2002 and 2017, thus extending the range of Caribbean islands with proven human cases of neuroangiostrongyliasis [12, 20, 44]. Given the potential lethality of neuroangiostrongyliasis, the medical community should therefore strongly consider the possibility of this infection in patients living in or returning from Martinique with eosinophilic meningitis.

In this study, neuroangiostrongyliasis cases occurred in two distinct epidemiological situations: three in infants less than 2 years old and five in patients over 11 years with mental diagnoses. The mode of transmission in infants was linked to poor living conditions with probable accidental consumption of contaminated snails or slugs when playing outside, as described on Mayotte, a French island in the Western Indian Ocean, in children under two years of age [18]. For adults, contamination was instead linked to intellectual disability associated with risky eating behaviour. In particular, pica syndrome may favour infection through the ingestion of gastropods usually not consumed as food in Martinique. No case was reported following consumption of raw paratenic hosts like shrimp, which is in contrast to the main source of infection in French Polynesia [35]. In our study, the diagnosis was initially unclear for all the patients and other kinds of helminthiases were initially suspected, as Martinique had never been reported as an endemic region for *A. cantonensis*. All but one of the patients were therefore treated with anti-helminthic drugs, although the efficacy and safety of albendazole or mebendazole for neuroangiostrongyliasis treatment remains controversial because of theoretical concerns that they may worsen the inflammatory response to dead and dying worms [5]. The four cases diagnosed after 2013 were also treated with corticosteroids, postulated to provide relief by reducing inflammation and thereby intracranial pressure and headache intensity [9, 43].

Comparison of the incidence of human neuroangiostrongyliasis in Martinique with neighbouring Caribbean Islands is straightforward. Indeed, cases were mainly reported in travellers returning from the Caribbean and it is likely that numerous autochthonous cases have not been reported in the scientific literature. Most Caribbean cases were reported in Cuba with several dozen cases since the 1980s [2, 36], mainly in the cities of Havana and Villa Clara [15, 16, 28, 37], and one case in a Swiss traveller returning from Cuba [7]. In Guadeloupe – another French West Indies island – four autochthonous cases were diagnosed (to our knowledge) between 1999 and 2017 in young children who may have been in contact with infected molluscs ([12] and unpublished data), corresponding to an annual incidence rate of 0.053 cases per inhabitant per year (95% CI [0.001–0.105]), close to that of Martinique. For the Dominican Republic, two suspected cases were reported in travellers returning to Europe [24, 41]. For Jamaica, twelve cases were diagnosed in adult travellers returning from Jamaica [38, 42], seven in autochthonous young children [19, 26] and one ocular case in a young woman [30]. No human cases have been reported in Grenada, the Bahamas, Haiti, and Puerto Rico, although *A. cantonensis* has been found in the environment on these islands [44]. It is noteworthy that the disease is also expanding in North and South America, in particular in the United States [27], Brazil [32], and some other South American countries [13, 44].

In this study, most cases occurred during the rainy season, during which snails abound, particularly the giant African snail, *Lissachatina fulica*, which was introduced to Martinique in 1989 [31] and is known elsewhere to act as an intermediate host of *A. cantonensis*. In Guadeloupe, *A. cantonensis* infection in *A. fulica* was 32.4% in 2014 [12]. No doubt other snail and slug species could also act as hosts as there are close to 90 non-marine mollusc species in Martinique [14, 23]. Numerous rodents have also been reported as potential definitive hosts of *A. cantonensis* worldwide [49]. Two species of rats*, Rattus norvegicus* (brown rat) and *Rattus rattus* (black rat), have been present in the territory since the late 18th century and probably play the role of definitive host of *A. cantonensis* in Martinique as they are the only rodent species in Martinique other than the mouse *Mus musculus*. No study has evaluated infection of rats with *A. cantonensis* in Martinique. In neighbouring Grenada [8, 10], Puerto Rico [4], Dominican Republic [45], Haiti [40], Jamaica [46], and Cuba [2], the proportion of infected *Rattus* spp. varies from 23.4% to 60.0% [2, 8], while the parasite appears to be absent in rats in Barbados [25].

Neuroangiostrongyliasis cases in Martinique seem particularly severe relative to other case-series reported in China and South-East Asia, with a higher mortality and sequelae rate. However, given the low number of cases, this must be confirmed as the number of neuroangiostrongyliasis cases increases in Martinique.

All cases were diagnosed by anti-*A. cantonensis* immunoglobulin detection in serum and/or CSF. Diagnosis was not performed by PCR as it was not available in Martinique or metropolitan France at the time of initial diagnosis [33] and no remaining CSF samples were available in our biobanks. In fact, molecular detection of *A. cantonensis* in CSF was developed in the early 2010s [48] and was only recently validated for clinical use [39]. The recent availability of a specific *A. cantonensis* PCR test in the French departments of South America and the Caribbean should improve the diagnosis of this disease in this region and encourage local authorities to undertake epidemiological studies on the intermediate and paratenic hosts and reservoirs, which should broaden our understanding of disease transmission in Martinique.
